# Investigation of the Water Damage Resistance and Storability of a SEBS-Modified Cold-Patching Asphalt Mixture

**DOI:** 10.3390/polym14235191

**Published:** 2022-11-29

**Authors:** Yuechao Zhang, Zirun Chen, Xiaoyuan Zhang, Yanhao Zhang, Xiaojun Zhu, Qinsong Li, Shuai Liu

**Affiliations:** 1 School of Civil Engineering and Architecture, Zhejiang Sci-Tech University, Hangzhou 310018, China; 2 Hangzhou Lushun Environmental Construction Co., Ltd., Hangzhou 311209, China

**Keywords:** CAM, mix design, water stability, SEBS, storability

## Abstract

At present, achieving good storability and water damage resistance remains challenging for cold-patching asphalt mixtures (CAMs). To address this issue, this study selects styrene–ethylene–butadiene–styrene copolymer (SEBS) and diesel as a modifier and diluent, respectively, to improve the water stability and storability of CAMs. The diesel oil content is determined through the Brookfield rotational viscosity test, and the modifier content is obtained through the Marshall stability test. With the empirical formula method, paper trail test, and modified Marshall test, mixed designs of CAMs modified with and without SEBS are established to determine the best cold-patching asphalt content. On this basis, the modification effect of SEBS is verified by comparing the test results of the modified and unmodified CAMs, and the water stability and Marshall stability tests are conducted before and after CAM storage, respectively. Results show that the optimum contents of SEBS and diesel oil are 7.5% and 40% of the base asphalt weight, respectively, and the best modified asphalt content is 4.6% of the mineral material weight in CAM. The Marshall residual stability and freeze–thaw splitting strength ratio of the 7.5% SEBS-modified CAM are increased by 20.1% and 15.7%, respectively, relative to the unmodified CAM, and the storage performance requirement of at least two months can be guaranteed.

## 1. Introduction

As one of the important components of infrastructure construction, highway construction has characteristics of long mileage, large volume, large resource occupation, and high energy consumption. At present, highway pavement surface materials mainly adopt hot-mix and hot-spread asphalt mixtures, and large amounts of these asphalt mixtures need to be produced in the construction process. The production of hot asphalt mixtures consumes much energy and resources, and it causes pollution to the surrounding environment [1]. Furthermore, due to the rDear Dr. Shirzad, equirement for environmental protection, the number of mixing stations in the construction of hot-mix and hot-pave asphalt mixtures in China is limited at present, which affects long distance transportation of these mixtures. Thus, cold-mix or warm-mix cold-paving asphalt mixtures have attracted increasing attention [2,3,4]. In view of these problems, people have paid increasing attention to the development and application of cold-paving asphalt mixtures with low energy consumption and storability in highway construction and maintenance.

Nowadays, cold-paving asphalt mixtures are mainly used as cold-patching materials in pavement maintenance engineering, and they are called cold-patching asphalt mixtures (CAMs). Scholars have conducted extensive work to investigate the performance of existing CAMs and develop new materials for them [5,6,7]. For example, Chen et al. [8] analyzed the influence of water-borne epoxy resin, a curing agent, and emulsified asphalt on water-borne epoxy resin-modified emulsified asphalt; then, they determined the reasonable formula of epoxy emulsified asphalt and put forward the improved Marshall experimental design method. Joni [9] studied the physical properties of two kinds of emulsified asphalt adhesives (cationic and anionic) used as paving mixture adhesives to evaluate the properties of the obtained emulsified asphalt mixture; the results of the Marshall stability test showed that the maximum Marshall stability of the cationic emulsified asphalt mixture is greater than that of the anionic emulsified asphalt mixture, and the optimum emulsification amount is 6.36%. However, due to the demulsification of emulsified asphalt, guaranteeing the long-term storage stability of cold-mix and cold-spread emulsified asphalt mixtures is difficult.

To fully improve the road performance and storage stability of CAMs, research has focused on solvent-based mixtures, with modifiers and diluents added to the base asphalt, to obtain CAMs with excellent performance. For instance, Tan et al. [10] established the raw material composition formula of CAMs through a series of studies and found that AC-13 gradation CAM has excellent freezing resistance with 70% SBS-modified asphalt, 30% kerosene, and 2% additive in the asphalt solution weight. Rezaei et al. [11] investigated the permanent deformation and moisture resistance of nine kinds of CAMs by using the Marshall stability test, indirect tensile strength test, and Hamburg rutting test. The results showed that the rutting resistance of CAMs with dense gradation is much higher than that of CAMs with open gradation. Moreover, the dust-to-binder ratio is highly related to the moisture sensitivity of dense-graded CAMs, and the percentage of coarse aggregates plays an important role in open-graded CAMs. Yuan et al. [12] studied the increase in the performance of CAMs with the extension of soaking time and the increase in cement content; they also put forward a new method to improve the moisture stability of CAMs with cement modification. Zhang et al. [13] presented a performance evaluation method for solvent-based cold-patching asphalt solutions and developed a new solvent-based cold-patching asphalt liquid (CAL) via the orthogonal design method; the CAL consisted of base asphalt, a diluent, a tackifier, a surfactant, and an anti-stripping agent. Huang et al. [14] determined the initial amount of CAM through infrared spectroscopy, four-component analysis, lying down method, and column core technology principle then optimized the formula by the orthogonal test design. The results showed that the road performance of self-made CAM is well verified. Xu et al. [15] evaluated the water-induced damage potential of CAM based on the surface-free energy theory and discovered that CAM has excellent water damage resistance when CAL contains alkali synthetic asphalt, rosin resin, diesel oil, and an anti-stripping agent and when the aggregate is composed of limestone and basalt. In addition, Pei et al. [16] analyzed the performance changes in CAL and its mixture at each stage and provided some suggestions for the preparation of CAM in cold weather. Gel Permeation Chromatography and Scanning Electron Microscope analysis could explain why the viscosity and strength of CAL increase after curing. The results showed that increasing the filler content can improve the deformation resistance of CAM.

Styrene–ethylene–butadiene–styrene (SEBS) copolymer, as a modifier obtained via styrene–butadiene–styrene block copolymer hydrogenation, is used in many fields because of its excellent physical and chemical characteristics. For instance, Gao et al. [17] prepared three kinds of nanocomposites and polypropylene/SEBS materials by adding media of different orders. An electrical branch aging test was then conducted with polypropylene, polypropylene/SEBS, and the nanocomposites. The results indicated that SEBS can promote the initiation and growth of the electric branch in polypropylene, and SiO_2_ inhibits the growth of polypropylene. Xue et al. [18] prepared a SEBS/h-SiO_2_ superhydrophobic composite coating on an aluminum alloy surface through the Czochralski method. The SEBS/h-SiO_2_ composite coating has a binary micro/nano rough structure and low surface energy, and it can form an air cushion when liquid droplets come into contact, thus showing excellent hydrophobicity.

Similarly, SEBS has also been used to improve the road performance of hot-mix asphalt mixtures. Ma et al. [19] determined the best content of SEBS through a road performance and fatigue resistance test, which showed that the late modulus and fatigue life of SEBS-modified asphalt mixtures are much better than those of base asphalt mixtures and gradually improve with the increase in content, indicating an excellent self-healing performance. Ke et al. [20] analyzed the physical properties, high-temperature storage stability, rheological properties, aging resistance, and micro-morphology of SEBS/organic montmorillonite-modified asphalt. The results showed that SEBS can considerably improve high- and low-temperature performance, but due to the poor compatibility between SEBS and asphalt, the modified asphalt exhibits serious phase separation during thermal storage.

However, investigations of the improvement in the water stability and storability of CAM after the addition of SEBS remain lacking. Therefore, this study analyzes CAL and its mixture modified with and without SEBS through a series of laboratory tests to obtain the mix design and performance of modified and unmodified CAM and evaluate the modification effect of SEBS on the water damage resistance and storage stability of CAM.

## 2. Materials

The raw materials of CAM used in this research included the modifier, diluent, base asphalt, and mineral aggregate [14]. The specific selected types and corresponding indicators of each material can be found in the following section.

### 2.1. Asphalt

Donghai brand 70# base asphalt was used as the asphalt binder, and it was purchased from Zhejiang Baoying Aisikai Materials Group Co., Ltd., Hangzhou, China. The detection indices met the requirements of the standard test methods and technical specifications for asphalt mixtures and pavements [21,22]. The main technical indices are shown in Table 1. 

### 2.2. Diluent

Diesel oil 0# was selected as the diluent of the base asphalt in this study, and the specific indicators are shown in Table 2.

### 2.3. Modifier

**Figure 1:**
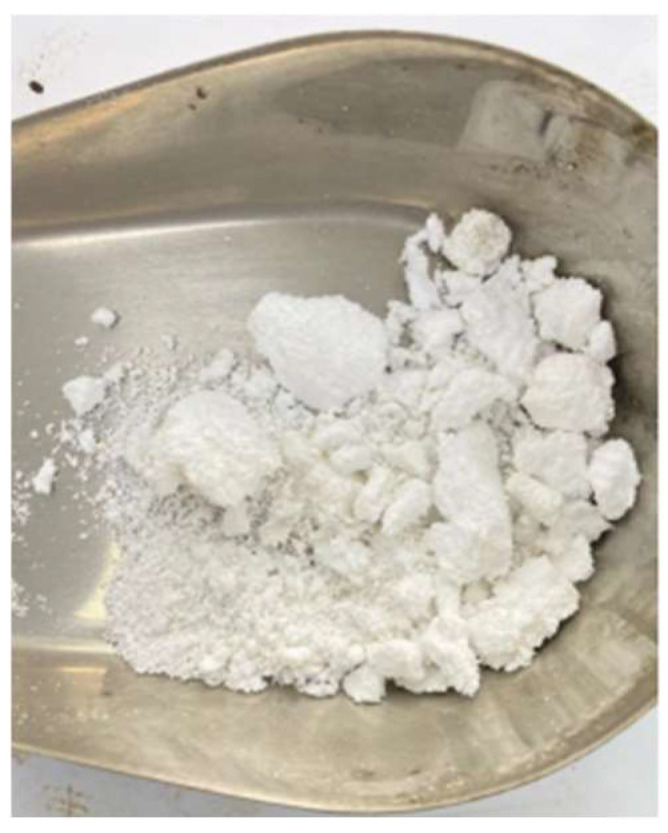
SEBS.

The modifier used in this study was SEBS, which was from Taicang Kelda Plastic Raw Materials Co., Ltd., Taicang, China. Its appearance is shown in Figure 1, and the specific technical indicators are shown in Table 3.

### 2.4. Mineral Materials

The aggregates and mineral powder were limestone, and the specific technical indices of the coarse and fine aggregates are shown in Table 4 and Table 5, respectively. All the technical indices of the aggregates and mineral powder met the requirements of relevant specifications in the Test Methods for Aggregate of Highway Engineering (JTG E42-2005) [23,24].

### 2.5. Gradation

**Figure 2:**
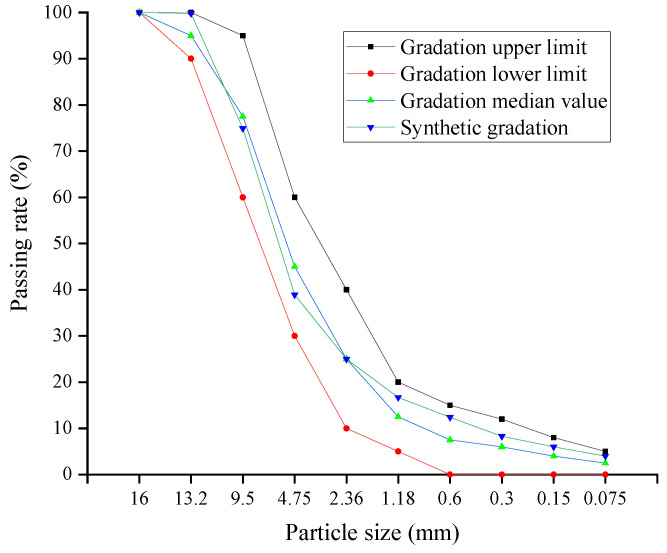
LB-13 gradation curve.

Referring to the Technical Specifications for Construction of Highway Asphalt Pavements (JTGF40-2004) [22], the gradation of CAM in this study was designed with LB-13 gradation in this specification, and the gradation curve is shown in Figure 2.

## 3. Test Methods

### 3.1. Brookfield Rotational Viscosity Test

The operation of the Brookfield rotational viscosity test is simple, and the viscosity of CAL can be characterized well; therefore, the construction workability and comprehensive performance of CAM can also be evaluated well. The Brookfield rotational viscosity test was used in this study to evaluate the viscosity of CAL and determine the appropriate content of the diesel diluent after adding different modifiers.

**Figure 3:**
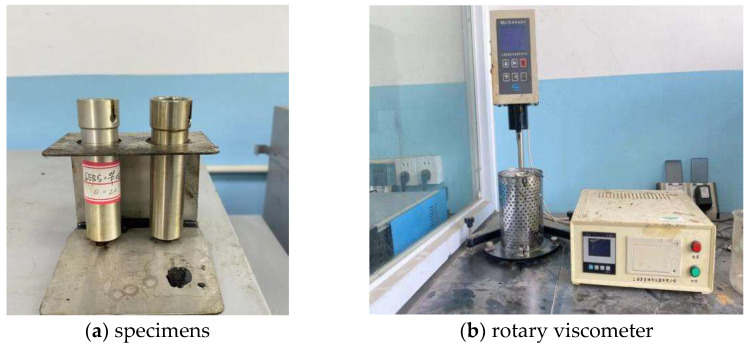
Brookfield rotational viscosity test process.

Referring to the Standard Test Methods of [21], the Brookfield rotational viscosity test was performed to reflect the late workability and fluidity of CAM after mixing. Generally, the Brookfield viscosity needs to be less than 3 Pa·s at a mixing temperature of 135 °C for hot-mix asphalt. Hence, considering the performance characteristics of CAM, the Brookfield viscosity is also less than 3 Pa·s at a mixing temperature of 60 °C for CAL. Moreover, according to research [25], when the Brookfield viscosity of CAL at 60 °C is about 2 Pa·s, the comprehensive performance of CAM is good. However, when the Brookfield viscosity of CAL is lower than 2 Pa·s, the workability of CAM is good, but its cohesiveness is poor. When the viscosity of CAL is too high, the fluidity of CAM is poor, which is not conducive to storage and paving. Therefore, selecting the rotational viscosity of 2–3 Pa·s at 60 °C is appropriate for CAL. The main steps of the Brookfield viscosity test conducted in this study were as follows:

(1) CAL preparation. The base asphalt was baked in a constant-temperature oven at 150 °C to a fluid state and then poured into a blender container, where the modifier and diluent weighed in proportion were added and fully blended with the asphalt for 20 min.
(2) The well-stirred CAL was poured into the specimen (two parallel specimens), as shown in Figure 3a.
(3) The CAL specimen was placed together with the rotor in a constant-temperature oven at 60 °C for 1.5 h.
(4) The specimen and rotor were taken out and placed in a rotary viscometer (Shanghai Changji Geological Instrument Co., Ltd., Shanghai, China), as shown in Figure 3b, where they were kept at 60 °C for at least 15 min.
(5) The test was initialized, and the viscosity change was observed and read when the two decimal places of viscosity were stable; it was read every 1 min three times in a row. The measured value was the average of the three readings. The same operation was performed for the other specimen.

### 3.2. Paper Trace Test

The paper trace test can evaluate the reasonable range of asphalt content in asphalt mixtures. This test method is similar to the test method used by Wang [25]. In the main processes of this test, about 800 g of the newly mixed asphalt mixture was placed on A4 white paper for about 3 min. Then, the mixture was removed, and the ink traces on the white paper were observed. The presence of very few ink traces meant that the asphalt content was too small and vice versa.

### 3.3. Marshall Stability Test

The Marshall stability test is mainly used for the mix design of asphalt mixtures and quality inspection of asphalt pavement construction. This test mainly refers to the Test Specification of [21]. In this study, first, the diluent and modifier were added to the base asphalt. Second, CAL was stirred thoroughly, and the mineral aggregate was placed in a mixer for preheating at 60 °C. Third, the stirred CAL was evenly poured into the mixer to mix the mixture and form CAM. Afterward, 1180 g of CAM was weighed and placed in the test mold of the Marshall electric compaction instrument at room temperature to mold specimens; both sides of the specimens were hammered 50 times. The specimen in the test mold was placed in an oven at 90 °C for 24 h in an upright way. Finally, the specimen was taken out and hammered 25 times. The specimen in the test mold was placed at room temperature for 24 h in an upright way and cured in a constant-temperature water tank at 60 °C for 30 min after demolding to carry out the Marshall test.

### 3.4. Water Stability Test

The water stability test mainly refers to the requirements of Test Specification [21] for related specimen molding and test loading.

#### 3.4.1. Immersion Marshall Test

**Figure 4:**
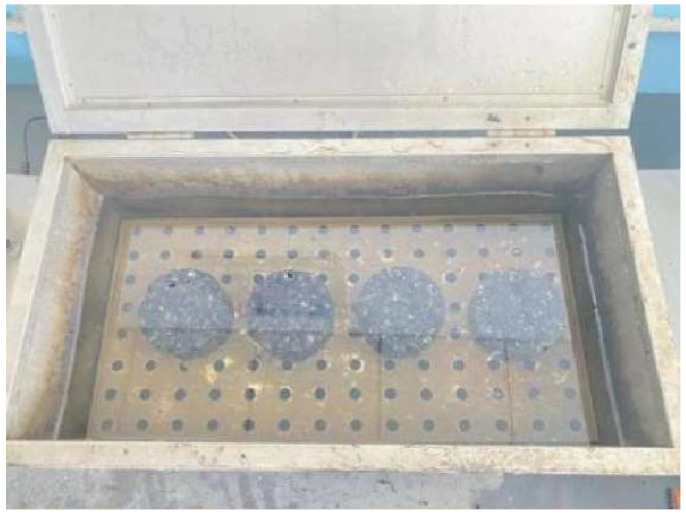
Immersed Marshall specimens.

The immersion Marshall stability test is used to test the ability to resist spalling when an asphalt mixture is subjected to water damage, and the feasibility of the mix design is tested by testing the water stability. The molding method of the immersion Marshall specimen is consistent with that of the Marshall specimen. In this study, eight specimens were prepared and divided into two groups, where each group had four specimens. Initial stability MS_1_ was directly tested after the first group of specimens was molded. However, testing the Marshall stability was difficult because the temperature in the 60 °C water bath was close to the mixing temperature of CAM, and the adhesion of the mixture was extremely low such that specimens could not be formed. Therefore, the stability MS_2_ of the second group of specimens was tested after specimens formed in a 25 °C water bath for 48 h. The immersed specimens are shown in Figure 4.

The residual stability, *MS*_0_, of CAM can be calculated as

(1) $$MS_{0}=\frac{MS_{2}}{MS_{1}}\times 100$$

where *MS*_2_ is the stability of Marshall specimens in unit kN after soaking for 48 h, *MS*_1_ is the stability of Marshall specimens after soaking for 30 min in unit kN, and *MS*_0_ is the residual stability of Marshall specimens in % after soaking for 48 h.

#### 3.4.2. Freeze–Thaw Splitting Test

**Figure 5:**
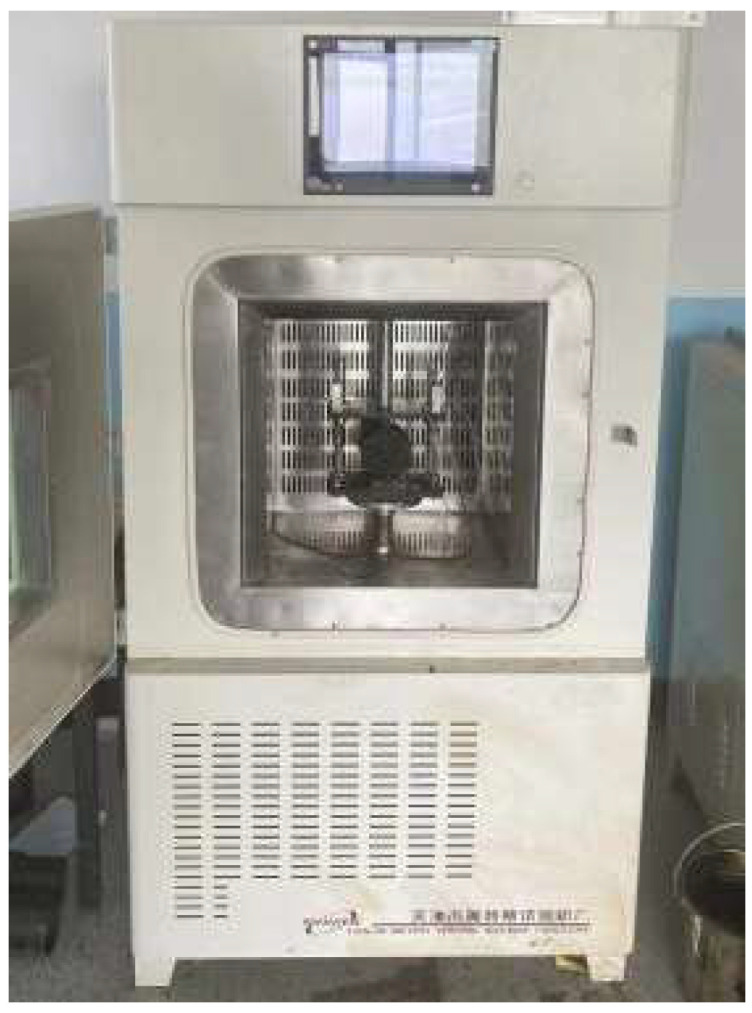
Splitting loading instrument of specimens.

The freeze–thaw splitting test is used to measure the strength ratio of an asphalt mixture before and after water damage in order to evaluate the water stability of the mixture. The test method of CAM refers to the freeze–thaw splitting test of hot-mix asphalt mixtures, and the specimen molding is similar to the Marshall test specimen molding method. The splitting loading instrument (produced by Tianjin Meters Testing Machine Factory, Tianjin, China.) is shown in Figure 5.

**Figure 6:**
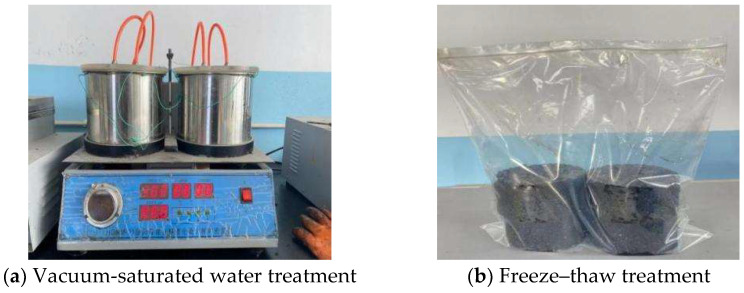
Freeze–thaw treatment of specimens.

For the test process, two groups of specimens were prepared, and each group had four specimens. One group of specimens was placed at room temperature for subsequent use after molding. The other group was treated with saturated water after molding and then placed in a plastic bag injected with 10 mL of water. The mouth of the bag was tightly sealed and placed in an environment of −16 °C ± 2 °C for 16 h, as shown in Figure 6. Then, the specimens were taken out and placed in a constant-temperature water tank at 45 °C for 24 h. Afterward, the two groups of specimens were placed in the constant-temperature water tank at 25 °C for 2 h, and the splitting test was carried out after the specimens were taken out.

The freeze–thaw splitting strength and the splitting strength ratio can be calculated as follows:

(2) $$R_{T1}=\frac{0.006287P_{T1}}{h_{1}}$$

(3) $$R_{T2}=\frac{0.006287P_{T2}}{h_{2}}$$

(4) $$TSR=\frac{\bar{R_{T2}}}{\bar{R_{T1}}}\times 100$$

where h_1_, *R*_*T*1_, and *R*_*T*1_ are the height, splitting load, and splitting tensile strength of the specimens without freezing–thawing in mm, N, and MPa, respectively; h_1_, *R*_*T*2_, and *R*_*T*2_ are the height, splitting load, and splitting tensile strength of the specimens subjected to freezing–thawing in mm, N, and MPa, respectively; $\bar{R_{T1}}$ and $\bar{R_{T2}}$ is the average of *R*_*T*1_ and *R*_*T*2_, respectively, and TSR is the strength ratio of the freeze–thaw splitting specimens in %.

### 3.5. Storage Stability Test

Relevant storage performance tests need to be performed to evaluate whether CAM can maintain the original workability and strength requirements after storage for a certain period. In this test, CAM after normal mixing was removed from the mixing pot and placed in a sealed bag for storage in a dark place for a certain period under a room temperature of 10–30 °C and relative humidity of 35–70%. After the specified storage time was reached, the bag was opened to observe the workability of CAM. Its grade standard is shown in Table 6.

Meanwhile, the CAM after storage was further molded via the Marshall test method, and its Marshall stability was measured to comprehensively evaluate the storage performance.

## 4. Test Results and Discussion

### 4.1. Determination of Diluent Content

**Figure 7:**
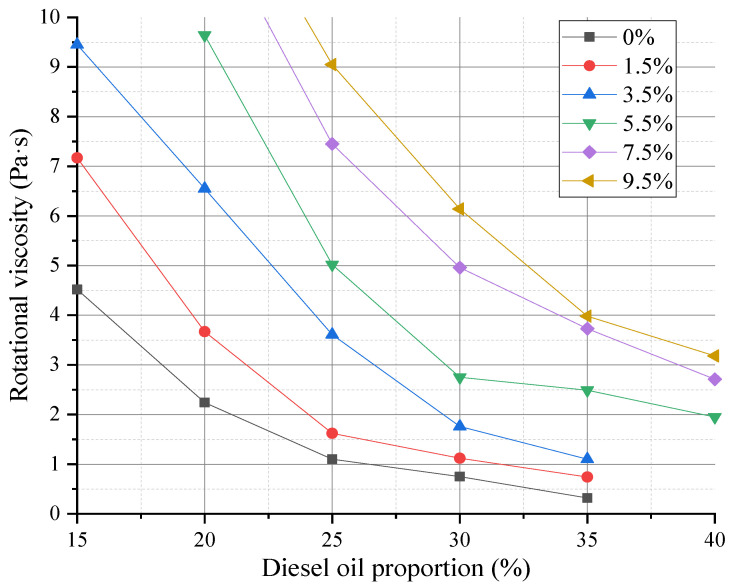
The effect of diesel oil on the rotational viscosity.

The average values of rotational viscosity with the different modifiers and diluents (calculated according to the weight percentage of the base asphalt) were obtained through the Brookfield rotational viscosity test. The dosage of the SEBS modifier was set to 0%, 1.5%, 3.5%, 5.5%, 7.5%, and 9.5% of the base asphalt weight. A viscosity test on CAL with different diesel diluents was performed for each modifier dosage. The specific results are shown in Figure 7.

The results in Figure 7 show that the Brookfield rotational viscosity gradually decreased with the increase in diesel oil content under the same SEBS content, but the decrease gradually slowed down, and the viscosity increased with the increase in SEBS content. To control the usage content of the diesel diluent, this study compared the viscosity of CAL at each modifier content with 40% diesel oil content. The results showed that the viscosity of CAL with 9.5% SEBS content was not between 2 and 3 Pa·s, whereas the other CAL with less than 9.5% SEBS content had a suitable diesel oil content range that can meet the viscosity requirement. The contents of diesel oil under 0%, 1.5%, 3.5%, 5.5%, and 7.5% SEBS contents were about 20%, 23%, 28%, 35%, and 40%, respectively. After determining the corresponding optimum diesel oil content with different SEBS contents in CAL, it was applied to determine the appropriate modifier dosage and the optimum asphalt content.

### 4.2. Determination of Modifier Content and Optimum Asphalt Content

#### 4.2.1. Preliminary Determination of Asphalt Content

(1) Results of the empirical formula method

The asphalt content (i.e., the ratio of modified asphalt and mineral material) in CAM was obtained according to the empirical formula method [27], as follows:

(5) P=0.021a+0.056b+0.099c+0.12d+1.2

where *P* represents the asphalt content of CAM in %, *a* is the weight percentage of particles greater than 2.36 mm in %, *b* is the weight percentage of particles between 0.3 and 2.36 mm in %, *c* is the weight percentage of particles between 0.075 and 0.3 mm in %, and D is the weight percentage of particles less than 0.075 mm in %.

Given the gradation of CAM in this test, in accordance with Formula (5), the asphalt content of LB-13 gradation CAM was calculated to be 4.6%.

(2) Paper trace test results

**Figure 8:**
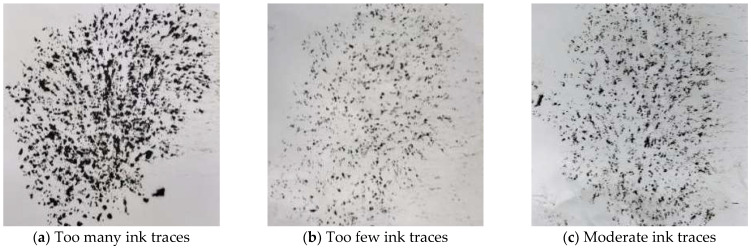
Comparison of ink traces on white paper for CAM with different asphalt contents.

After the asphalt content in CAM under a given gradation was preliminarily obtained using the empirical formula method, the paper trace test was further performed in a certain range of 4.6% asphalt content to compare with the ink traces on white paper and to verify the rationality of the used asphalt content. The typical results of the paper trace test with the CAM with 7.5% SEBS as an example are shown in Figure 8. Figure 8a–c correspond to the presence of ink traces on white paper for the CAM with 5.2%, 4.0%, and 4.6% asphalt content, respectively.

As shown in Figure 8, compared with the moderate ink traces on white paper for CAM with 4.6% asphalt content, the oil content for CAM with 5.2% asphalt content was too much, indicating that the amount of asphalt in CAM was too much. The oil content for CAM with 4.0% asphalt content was too little, showing that the amount of asphalt in CAM was too little. The paper trace test results for the other SEBS contents were similar to those with 7.5% SEBS content, so the range of asphalt content in CAM with different SEBS contents was determined to be 4.0–5.2%.

#### 4.2.2. Determination of Modifier Content

**Figure 9:**
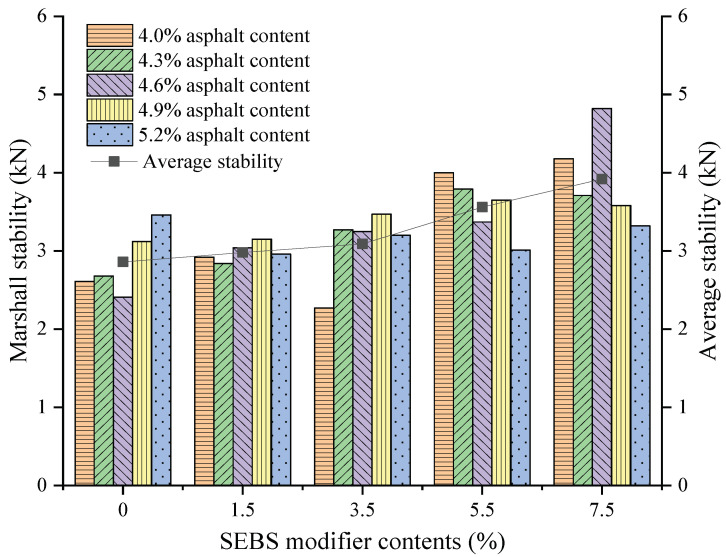
Marshall stability of modified CAM with different SEBS contents.

On the basis of the asphalt content range determined by the above-mentioned CAM with different SEBS contents, a Marshall stability test under each SEBS content was conducted within the determined asphalt content range to obtain the average Marshall stability. Then, the optimal content of the SEBS additive was determined, and the results are shown in Figure 9, where the bar and line graphs correspond to the results for Marshall stability and average Marshall stability of five asphalt contents at each SEBS modifier content, respectively. The average Marshall stability increased with the increase in SEBS content. Compared with the result without the modifier, the results of 1.5%, 3.5%, 5.5%, and 7.5% SEBS contents increased by 0.12, 0.23, 0.7, and 1.06 kN, respectively. The stability under 7.5% SEBS content was the highest, so the modifier content in CAM was selected as 7.5%.

#### 4.2.3. Determination of the Optimum Asphalt Content

**Figure 10:**
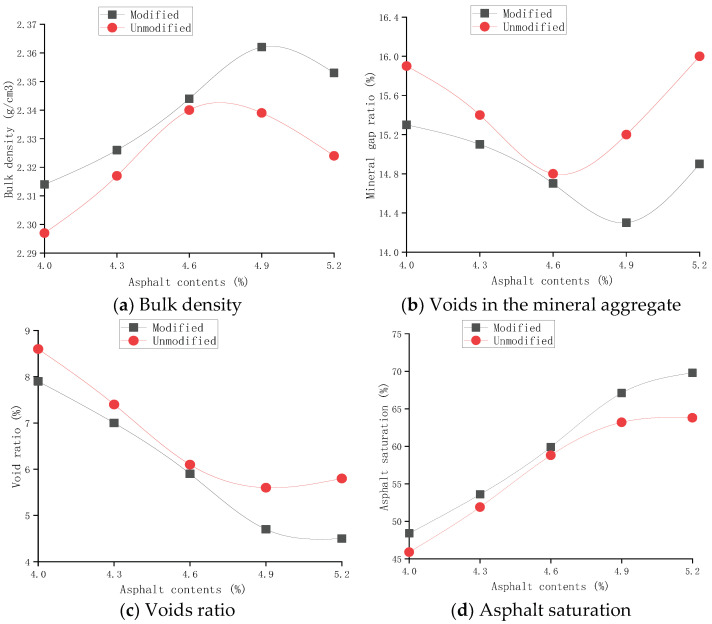

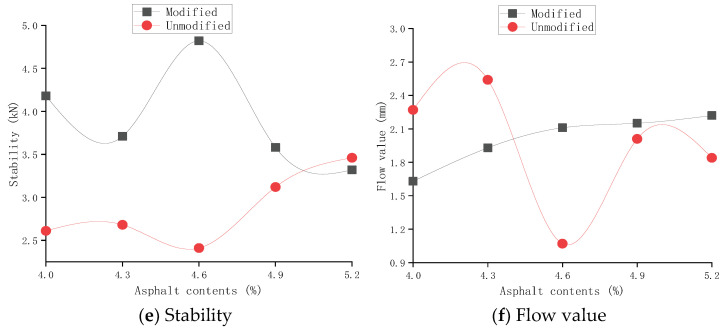
Relationship between asphalt content of CAM and Marshall indices.

After determining the reasonable content of the modifier in a certain asphalt content range, the modified Marshall method was further used to determine the optimum asphalt content [28]. The index results of CAM with 7.5% and without SEBS were compared, as shown in Figure 10.

According to the results in Figure 10, the asphalt content corresponding to the maximum bulk density, the maximum stability, and the target void ratio (or median) was obtained as a_1_, a_2_, and a_3_, respectively. Then, the optimum asphalt content (OAC) of CAM was calculated as OAC = (a_1_ + a_2_ + a_3_)/3, where the OAC of CAM with 7.5% SEBS and without a modifier was 4.6% and 4.8%, respectively.

### 4.3. Results and Discussion of Water Stability

On the basis of the mix design of the CAM obtained above, this study further evaluated the water stability of CAM with 7.5% SEBS and without a modifier to verify the water damage resistance modification effect of CAM with SEBS.

#### 4.3.1. Immersion Marshall Test Results

**Figure 11:**
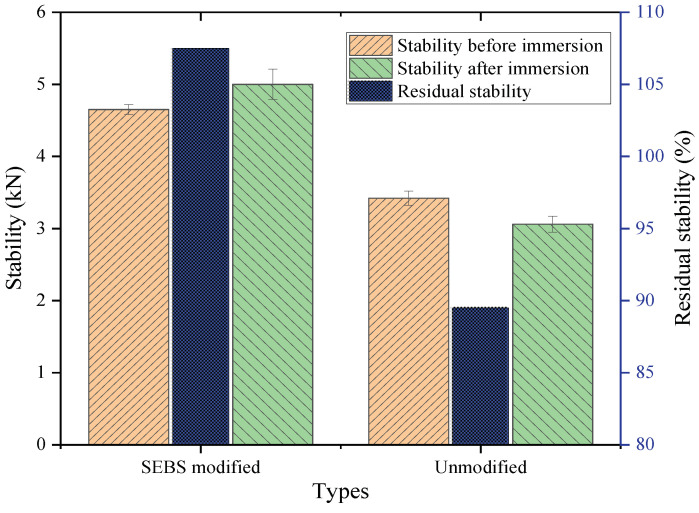
Results of the immersion Marshall test.

Figure 11 indicates that the stability of the SEBS-modified CAM specimen before immersion was 4.65 kN, and that of the specimen without a modifier was 3.42 kN; the tested result of the former was 1.23 kN larger than that of the latter. After the specimens were immersed in a water bath for about 48 h at 25 °C, the stability of the modified specimen became 5 kN, indicating an increase of 0.35 kN compared with the value for the un-immersed specimen. The stability of the unmodified specimen after immersion was 3.06 kN, which was 0.36 kN lower than that of the un-immersed specimen.

This phenomenon may be due to the fact that although the immersed specimen reduced the CAL viscosity and led to a reduction in specimen strength, the diesel oil content in the modified CAM was twice as much as that in the unmodified CAM (i.e., the former’s content was 40%, and the latter’s was 20%), and the diesel oil loss of the modified specimen was larger than that of the unmodified specimen in the immersion process. For the modified specimen, the increase in CAL viscosity caused by diesel oil loss had a greater effect than the decrease in CAL viscosity caused by water damage after immersion, so the specimen’s stability was greater than that before immersion. 

In addition, the residual stability of the modified specimen at 25 °C was 107.5%, and that of the unmodified specimen was 89.5%. Compared with the latter, the former increased by nearly 20.1%, indicating that the water stability of the specimen was improved after adding SEBS to CAM, and the modification effect was remarkable.

#### 4.3.2. Freeze–Thaw Splitting Test Results

**Figure 12:**
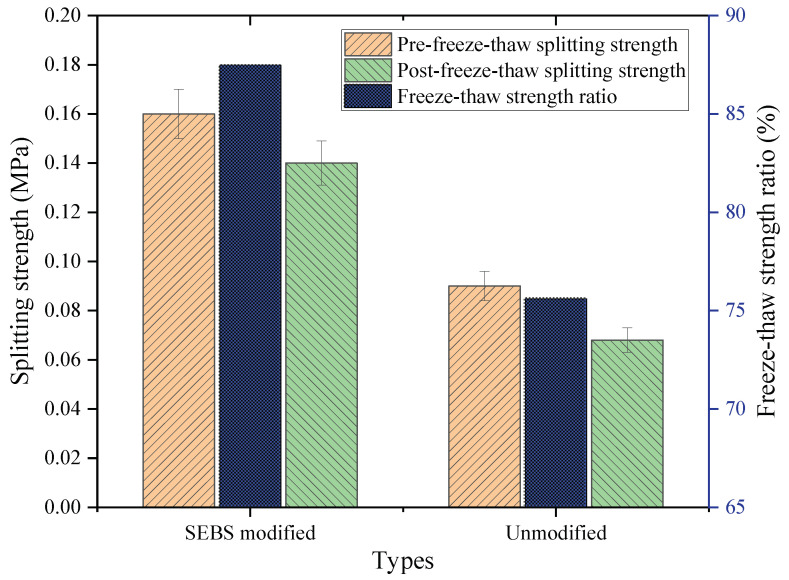
Results of the freeze–thaw splitting test.

Figure 12 shows that the unfrozen splitting strength of the SEBS-modified CAM specimen was 0.16 MPa, and that of the unmodified CAM specimen was 0.09 MPa. The former was 1.78 times the latter. After the freeze–thaw cycles, the splitting strengths of the two specimens decreased. The former’s splitting strength was 0.14 MPa, and that of the latter was 0.068 MPa; the former was still 2.06 times the latter. This result indicates that whether before or after the freeze–thaw action, the strength of the CAM with SEBS was considerably improved compared with that of the CAM without a modifier. Further illustrating the modified effect of water stability, the freeze–thaw splitting strength ratio of the modified specimen was 87.5%, and that of the unmodified specimen was 75.6%. Thus, the splitting strength ratio of the SEBS-modified specimen was higher than that of the unmodified specimen, namely, the former increased by about 15.7% compared with the latter. This result further indicates that the SEBS-modified CAM had a remarkable effect.

### 4.4. Storage Performance

**Figure 13:**
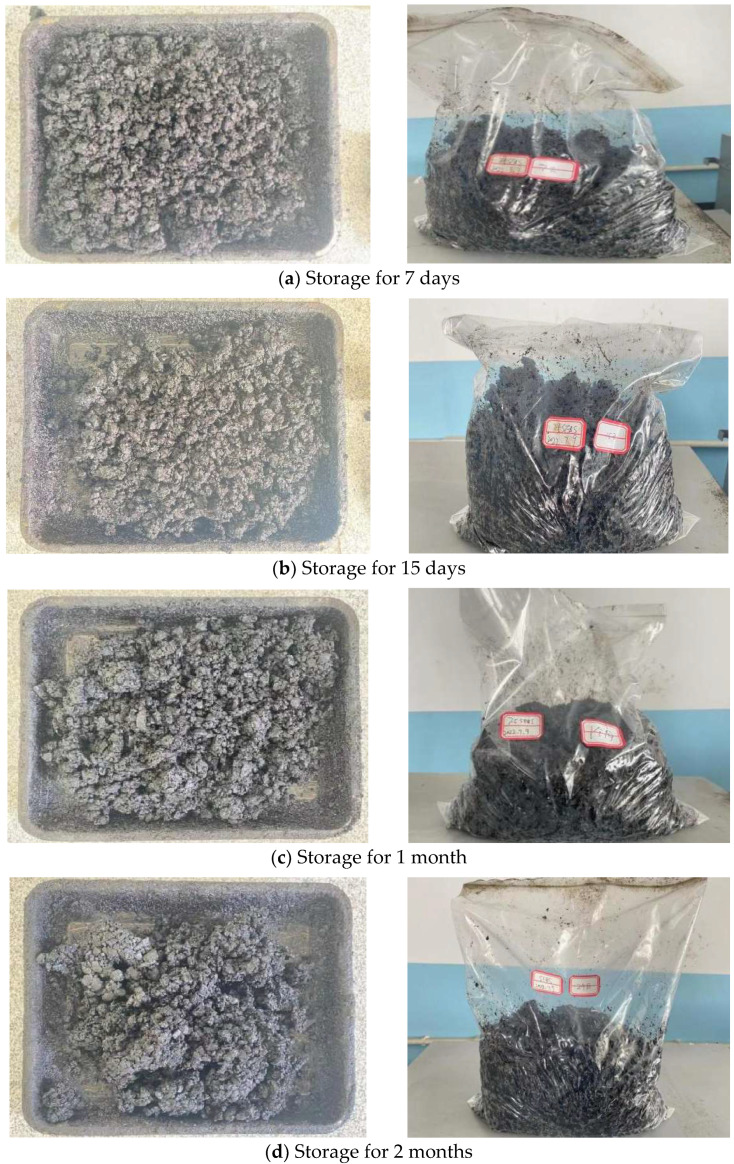
State of the modified CAM at different storage times.

In addition to the evaluation of the water damage resistance of CAM, this study also compared the construction workability of CAM with and without the 7.5% SEBS content modifier after storage for 0 days, 7 days, 15 days, 1 month, and 2 months. For the state of CAM after storage for a certain period, typical results of the modified CAM are shown in Figure 13.

**Figure 14:**
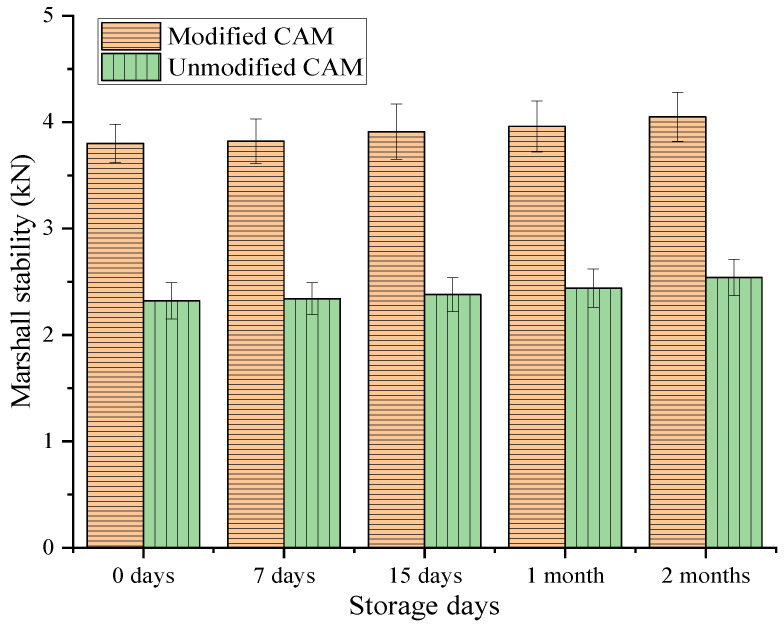
Results of the storage performance of the modified and unmodified CAM.

As shown in Figure 13, the SEBS-modified CAM had clear particles and no caking after storage for 7 days, that is, its workability grade was 1. After storage for 15 days, 1 month, and 2 months, CAM exhibited a small amount of caking, but it could be scattered, that is, its workability grade was 2. Meanwhile, the storage and workability grades of the unmodified CAM were similar to those of the SEBS-modified CAM. Therefore, we conclude that CAM does not exhibit agglomeration during short-term storage, but a small amount of agglomeration will occur with the increase in storage time. However, the agglomeration can be stirred and scattered by a shovel, so it has little impact on subsequent usage. In addition, the Marshall stability of the modified and unmodified CAM after storage was tested, and the results are shown in Figure 14.

After storage for 0 days, 7 days, 15 days, 1 month, and 2 months, the stability of the SEBS-modified CAM was 3.8, 3.82, 3.91, 3.96, and 4.05 kN, respectively, and the respective results of the unmodified CAM were 2.32, 2.34, 2.38, 2.44, and 2.54 kN. Therefore, whether for the modified or unmodified CAM, the stability increased with the increase in storage time, but the increase was small. For example, during two months of storage, the stability of the modified and unmodified CAM increased by only 6.6% and 9.5% compared with the results for 0 days of storage, respectively. This result may be due to the gradual volatilization of the diluent during storage, which increased the CAL viscosity. However, the volatilization amount was small, so CAL had a limited increase in viscosity. According to the comparison of the stability of the two types of CAM, the result of the modified CAM was much higher than that of the unmodified CAM. For instance, when stored for two months, the stability of the former was 1.59 times that of the latter. To sum up, the CAM with the SEBS modifier showed good storage performance, and it could meet construction workability and stability requirements after two months of storage.

## 5. Conclusions

Through a series of laboratory tests and analyses, the mix design, water stability, and storage stability of pristine and SEBS-modified CAM specimens were investigated and the following conclusions were obtained.

(1) The rotational viscosity test conducted at 60 °C, the Marshall stability test, and the paper trail test revealed that the SEBS modifier and diesel diluent in the modified CAL were 7.5% and 40% of the base asphalt weight fraction, respectively, and the optimum asphalt content of the modified CAM was 4.6%. Meanwhile, the diesel diluent in the unmodified CAM was 20%, and the optimum asphalt content of the unmodified CAM was 4.8%.
(2) The immersion Marshall test and the freeze–thaw splitting test showed that the residual stability and freeze–thaw splitting strength ratio of the modified CAM were 107.5% and 87.5%, respectively, which were much higher than those of the unmodified CAM (89.5% and 75.6%) and could meet the specification requirements. Thus, compared with the unmodified CAM, the SEBS-modified CAM had better water stability. Adding the SEBS modifier improved the water stability of CAM.
(3) The storage performance test revealed that the Marshall stability of the modified CAM was higher than that of the unmodified CAM by more than 1 kN within two months of storage. Adding SEBS improved the stability of CAM. In conclusion, SEBS-modified CAM can be stored for at least two months, and its stability will increase with the increase in storage time. However, the increase will be small, and its workability will not change much. Thus, this mixture has good storage performance.

## Figures and Tables

**Table 1:** Test results of the base asphalt index.

Test Item	Requirements	Test Result	Test Methods
Penetration (25 ℃, 100 g, 5 s) 0.1 mm	60–80	66	T0604
Penetration index	−1.5~+1.0	−0.1	
Softening point (ring and ball method) (℃)	≥46	48	T0606
10 ℃ Ductility (cm)	≥20	32	T0605
15 ℃ Ductility (cm)	≥100	100	
Wax content (distillation method) (%)	≤2.2	1.8	T0615
60 ℃ Dynamic viscosity (Pa·s)	≥180	188	T0620
Flashing point (℃)	≥260	280	T0611
Solubility (%)	≥99.5	99.8	T0607
After the rolling thin film oven test (TFOT)			
Mass loss (%)	≤0.8	0.06	T0609
Penetration ratio (25 ℃) (%)	≥61	68	T0604
Residual ductility (10 ℃) (cm)	≥6	6.5	T0605

**Table 2:** Physical properties of diesel oil.

Properties	Unit	Results
Flashing point	°C	64
Density	g/cm^3^	0.83
Kinematic viscosity (20 °C)	mm^2^/s	7.5
Condensation point	°C	−5

**Table 3:** Technical indicators of SEBS.

Properties	Unit	Typical Values
Structure	-	linear
Ethylene content	%	33
Tensile strength at break	MPa	25
Tensile stress	MPa	6.0
Tensile elongation at break	%	500
Viscosity of 10% solution at 25 °C	Pa·s	1500

**Table 4:** Technical index of the coarse aggregate.

Index	Unit	Technical Indicators	Test Methods
Apparent specific density	-	2.70	T0304
Bulk specific density	-	2.61	T0308
Crushed value	%	19.2	T0316
Flat and elongated particle content	%	13.9	T0312
Less than 0.075 mm particle content	%	0.36	T0310

**Table 5:** Technical index of the fine aggregate.

Index	Unit	Technical Indicators	Test Methods
Apparent specific density	-	2.74	T0328
Sediment percentage	%	0.6	T0333
Sand equivalent	%	79	T0334
Soundness	%	5	T0340

**Table 6:** Reference standard of the workability grade of CAM [26].

Condition	Grade
Mixture has clear particles and no agglomeration	1
A few lumps but can be scattered	2
Many small clumps but can be scattered	3
Many clusters and large volume; cannot be scattered	4

## Data Availability

The data presented in this study are available on request from the corresponding author.
